# Preventive treatment of tripdiolide ameliorates kidney injury in diabetic mice by modulating the Nrf2/NF-κB pathway

**DOI:** 10.3389/fphar.2025.1492834

**Published:** 2025-03-19

**Authors:** Bo Yuan, Dan Jia, Baoshan Gao

**Affiliations:** Department of Urology, The First Hospital of Jilin University, Changchun, China

**Keywords:** diabetic nephropathy, tripdiolide, oxidative stress, inflammation, intestinal microbiota

## Abstract

**Introduction:**

Although tripdiolide has demonstrated a protective role in lupus nephritis, its potential therapeutic and preventive effects on diabetic kidney injury remain inconclusive.

**Methods:**

In this study, a diabetes mice model was used to evaluate the effect of preventive treatment of tripdiolide on the kidney. The study assessed diabetes related factors levels, while comparing kidney pathological changes, alterations in intestinal microbiota composition, oxidative stress and inflammation in kidneys, validating cytokine expression and protein pathway activation.

**Results:**

The experiment demonstrated that tripdiolide preventive treatment effectively suppressed the hyperglycemia and elevated hemoglobin level, attenuated the concentrations of creatinine and blood urea nitrogen, mitigated histopathological alterations in the kidney, and alleviated inflammatory cell infiltration. Tripdiolide regulated intestinal microbiota in diabetes mice and affected the abundance of *Allobaculum*, *Dubosella*, and *Prevotella*, and the differential metabolic pathways primarily revolve around ubiquinol biosynthesis and menaquinol biosynthesis. Tripdiolide treatment significantly attenuated renal oxidative stress and inflammation in diabetic mice, as evidenced by the upregulation of nuclear factor erythroid 2-related factor 2 (Nrf2), heme Oxygenase-1, and the downregulation of phosphorylated nuclear factor-κB (P-NF-κB), and NOD-like receptor protein 3. Experiments performed in RAW264.7 cells demonstrated the effect of tripdiolide.

**Discussion:**

Tripdiolide may play a protective role in hyperglycemia induced kidney injury by changing the composition of intestinal microorganisms, regulating Nrf2/NF-κB pathway activation, and inhibiting oxidative stress and inflammatory reaction. This study contributes scientific evidence that can inform the development of preventive therapeutic approaches for diabetic nephropathy.

## 1 Introduction

Diabetes is a long-term health condition characterized by consistently elevated blood sugar levels, known as hyperglycemia, which can give rise to numerous complications. Annually, diabetes accounts for over 1 million fatalities and imposes a substantial health burden (Khan et al., 2020; Liu P. et al., 2024). Persistent hyperglycemia can lead to severe microvascular disease, causing damage to the kidneys, eyes, nerves, and teeth. Diabetic nephropathy, being the prevailing complication of diabetes, stands as the primary contributor to end-stage kidney disease (Filippatos et al., 2021; Samsu, 2021). More than 30% of diabetes patients are complicated with chronic kidney disease as the disease progresses, and about 35% of end-stage kidney disease patients are related to diabetes (Chaudhuri et al., 2022). The projected number of deaths attributed to chronic kidney disease is anticipated to reach an estimated 2.2 million individuals in the most favorable situation and approximately 4 million people in the least favorable situation by 2040 (Foreman et al., 2018).

In patients with diabetes, various mechanisms, including stimulation of the renin-angiotensin-aldosterone pathway and buildup of advanced glycation end products, contribute to kidney damage (Hu et al., 2023). Angiotensin II and aldosterone are the primary kidney toxins that can induce oxidative stress response, pro-inflammatory effects, and promote fibrotic effects at both cellular and molecular levels (Villarreal et al., 1993; Pacurari et al., 2014). Reactive oxygen species (ROS) production is increased as a consequence of excessive accumulation of advanced glycation end products (Liu et al., 2021), which subsequently induces alterations in mitochondrial permeability and disruption of mitochondrial membrane potential, ultimately leading to the apoptosis of mesangial cells (Jin et al., 2019). ROS can activate NOD-like receptor protein 3 (NLRP3) through the nuclear factor-κB (NF-κB) pathway (Rajamäki et al., 2016). Advanced glycation end products have the ability to bind with toll-like receptor 4 (TLR4), which activate the inhibitor of NF-κB (IκB)/NF-κB complex interactions and subsequent promotion of NLRP3 and pro-interleukin (IL)-1β transcription (Hodgkinson et al., 2008; Ogata et al., 2013). The interaction between glucose and lipid metabolism disorders and hemodynamic changes can induce a large amount of ROS production, further activating cellular signaling pathways, causing inflammatory reactions, autophagy, fibrosis, Reinforcing the pathological alterations and functional irregularities at an accelerated pace that lead to diabetic kidney disease (Ma et al., 2023). Targeting oxidative stress and inflammatory response has emerged as a prominent research area in the management of diabetes and diabetic nephropathy (Caturano et al., 2023; Rayego-Mateos et al., 2023).

The role of intestinal microbiota in the pathogenesis and prevention of diabetic nephropathy is garnering increasing attention (Yu et al., 2024). Patients with diabetic nephropathy exhibit imbalances in the structure, quantity, and variety of intestinal microbial communities, abundance, and diversity of intestinal microbiota (Kikuchi and Abe, 2020). Compared to healthy controls, patients with diabetes exhibited significantly elevated levels of *Proteobacteria*, *Verrucomicrobia*, and *Fusobacteria* in intestinal microbiota (Salguero et al., 2019). The intestinal microbiota of stage 3 or stage 4 diabetic nephropathy patients exhibits a higher abundance of *Haemophilus*, *Megalococcus*, *Veillonella*, and *Anaerostipes* (Du et al., 2021). The dysbiosis of intestinal microbiota in diabetic nephropathy patients has a connection to elevated levels of toxins, heightened inflammation, impaired intestinal barrier function, and decreased short-chain fatty acids (Kikuchi and Abe, 2020). *Prevotella* and *Bacteroides* can increase the toxins producing (Das et al., 2021), metabolized toxins are transferred to the bloodstream, exacerbating kidney injury (Lv et al., 2022). The increase in *Proteus* abundance promotes inflammation and reduces the presence of short-chain fatty acids producing bacteria in patients with diabetic nephropathy, potentially constituting a significant mechanism underlying the progression of renal complications in individuals with diabetes (Salguero et al., 2019; Stavropoulou et al., 2021). The transplantation of microbiota has shown effective demonstration retard the progression of diabetic nephropathy by enhancing intestinal microbiota stability and reducing toxin production (Bock et al., 2021; Shang et al., 2022). Microbiota transplantation improves glomerular injury induced by streptozotocin (STZ) in rats with diabetes (Lu et al., 2021). In type 2 diabetes mice, microbiota transplantation enhanced glucose tolerance and reduced insulin resistance, while mitigating damage to the islets (Wang et al., 2020). Modulation of the intestinal microbiota contributes to ameliorating the initiation and development of diabetic nephropathy.

The current evidence supports that optimal control of glycemic levels and hypertension management remains the sole proven primary preventive measure for diabetic nephropathy patients (Elsayed et al., 2024). Despite the adjunctive use of targeted therapies alongside conventional hypoglycemic and antihypertensive treatments, regrettably, a significant number of patients continue to advance towards end-stage renal disease (Zhang et al., 2020). Therefore, there is an urgent imperative for the advancement of innovative therapeutic approaches aimed at enhancing the therapeutic outcomes of diabetic nephropathy through a more targeted approach. Therapeutic interventions focused on prevention may offer superior benefits in reducing diabetes-related kidney injury compared to reactive approaches (El Din et al., 2017). Natural compounds have demonstrated tremendous potential in drug development due to their unique structures and diverse biological activities. From traditional herbal medicine to modern drug discovery, natural compounds have consistently served as a vital source for the development of novel therapeutics. Tripdiolide serves as the primary active ingredient found in *T. wilfordii* Hook F, a traditional Chinese herbal remedy, and tripdiolide exhibited protective effect in lupus nephritis in mice (Tao et al., 2008). *T. wilfordii*, while inherently toxic, offers a valuable opportunity to identify non-toxic or low-toxicity compounds for the treatment of diabetic nephropathy. Investigating the compounds of *T. wilfordii* not only helps avoid adverse effects but also contributes to a clearer understanding of their mechanisms of action. However, the preventive and therapeutic effects of tripdiolide in diabetic kidney disease, in addition to underlying mechanisms, remain unexplored.

In this study, a type 2 diabetes mice model was established to evaluate the protective effect and underlying mechanism of tripdiolide on kidney injury in diabetes mice through preventive treatment with tripdiolide.

## 2 Materials and methods

### 2.1 Animal experimental protocol

Twenty-four male C57BL/6 mice (20–22 g, 8 weeks old) purchased from Liaoning Changsheng Biotechnology Co., Ltd. (SCXK [LIAO]-2020-0001, Liaoning, China) were provided with food and water that had undergone sterilization, while being maintained under consistent temperature and humidity conditions. Additionally, they were subjected to a light/dark cycle lasting for 12 h each. The experiments involving animals were carried out with international ethical standards for animal experimentation (ARRIVE guidelines.) and approved by the Institutional Animal Ethics Committee of Jilin University (SY202312014).

After 7 days acclimatization, mice were divided into three groups in a random manner, control group (Ctrl) mice (n = 8 per group) and diabetic model group (Model) mice (n = 8 per group) were intragastrically (i.g.) administered 5 mL/kg normal saline (NS with 0.2% sodium carboxymethyl cellulose), and tripdiolide-treated diabetic model mice (n = 8 per group) were administered (i.g.) 200 μg/kg tripdiolide (dissolved in NS with 0.2% sodium carboxymethyl cellulose) (CAS: 38647-10-8, Beijing Wokai Biotechnology Co., LTD., Beijing, China) daily for 10 weeks. During this period, Ctrl mice were fed with normal chow diet (Liaoning Changsheng Biotechnology Co., Ltd.), while other mice were fed high-fat diet (comprising 60% of total calories from fat, 20% from protein, and 20% from carbohydrates; Xiao Shu You Tai Biotechnology Co., Ltd., Beijing, China). In the initial 5 days of the fifth week, Ctrl mice were intraperitoneally injected with NS, while other mice received an intraperitoneally injected of 1% Streptozotocin (STZ, #S25467, Shanghai yuanye Bio-Technology Co., Ltd., Shanghai, China) citrate buffer solution (40 mg/kg) once a day to induce diabetes.

### 2.2 Measurement of blood indicators and tissue collection

After the final administration, the body weight, 24 h food intake level and 24 h water intake level were measured. The blood glucose was assessed utilizing a blood glucose meter (Sinocare Inc., Changsha, China) and blood HbA1c was measured using A1CNow Self Check (Sinocare Inc.) after fasting for 10 h. Then, blood was collected from mouse tail vein, stood at 25°C for 30 min and centrifugation at 3,000 r/min for 10 min to obtain the serum sample. After administering carbon dioxide inhalation to induce euthanasia in the mice, the kidney, liver, heart, spleen, pancreas, and cecal contents were immediately collected and weighted. Organ indices were calculated as follows:

$$\text{Organ index }\left(\%\right)=\text{organ weight }\left(g\right)\;/\text{ body weight }\left(g\right)\times 100$$

Some kidneys were preserved in a 4% paraformaldehyde solution for future histopathological analysis. The remaining tissues, serum, and cecal contents were stored at a temperature of −80°C.

### 2.3 The effects of tripdiolide in RAW264.7 cells

RAW264.7 (#TIB-71) cell line was obtained from American Type Culture Collection (ATCC, MD, United States) and cultured in complete RPMI 1640 medium (#11875093, Gibco, Thermo, MA, United States) in a complete humidity incubator with CO_2_/air (5%/95%) at 37°C. RAW264.7 cells (5 × 10^5^ cfu/mL) were seeded into 96-well plates and cultured for 12 h. The cells were then treated with 0, 25 and 100 mg/L tripdiolide and incubated for additional 12 h. After incubation, 100 μL Lipopolysaccharides (LPS, #S55571, Shanghai yuanye Bio-Technology Co., Ltd.) was added for 24 h.

### 2.4 Histopathological examination

According to previous study (Qu et al., 2023), the kidney tissues of each group of mice (n = 3) were chosen in a random manner and then immersed in 4% paraformaldehyde (#W12492, Shanghai yuanye Bio-Technology Co., Ltd.) for a duration of 48 h. Afterwards, a series of ethanol (#10009159), xylene (#10023418), and paraffin (#69018961) (Sinopharm Chemical Reagent Co., Ltd., Shanghai, China) solutions were used to progressively process the kidneys before embedding them in paraffin. Additionally, 5 µm sections of paraffin-embedded kidneys were meticulously deparaffinized using xylene and a gradient concentration of ethanol, subsequently subjected to staining using hematoxylin and eosin (H&E) Stain Kit (#G1120, Solarbio, Beijing, China). Ultimately, the sections underwent microscopic examination (ECLIPSE E100, Nikon, Tokyo, Japan). With reference to prior studies and adapting as necessary (Tak et al., 2013; Yamanouchi et al., 2018), extracellular matrix deposition and inflammatory cell infiltration were scored according to the scoring system in Table 1.

**TABLE 1:** Histological scores of kidney sections.

	Score	Ctrl (n = 5)	Model (n = 5)	TDe (n = 5)
Extracellular matrix deposition	0	3	—	—
	1	2	—	2
	2	—	2	2
	3	—	3	1
Average score		0.4	2.6	1.8
Inflammatory cell infiltration	0	4	—	—
	1	1	1	3
	2	—	1	1
	3	—	3	1
Average score		0.2	2.4	1.6

Range 0–3 for extracellular matrix deposition: 0, no extracellular matrix deposition; 1, mild extracellular matrix deposition; 2, moderate extracellular matrix deposition; 3, severe extracellular matrix deposition in glomeruli. Range 0–3 for inflammatory cell infiltration: 0, no inflammatory cell infiltration; 1, mild inflammatory cell infiltration; 2, moderate inflammatory cell infiltration; 3, severe inflammatory cell infiltration in glomeruli. TDe, tripdiolide.

### 2.5 The cytokines analysis

The kidney tissues mixed with NS at a ratio of 1:10 (w: v). After homogenization, centrifuged at 10000 r/min to obtain the supernatant as the kidney samples for testing. Protein concentration was quantified using the BCA assay kit (#23227, Thermo). The standard detection kits were employed for the quantification of serum insulin (#H203-1-1), blood urea nitrogen (BUN) (#C013-2-1), creatinine (Cr) (#C011-2-1), kidney ROS (#E004-1-1), glutathione peroxidase (GSH-Px) (#A005-1), malondialdehyde (MDA) (#A003-1), superoxide dismutase (SOD) (#A001-3) (Nanjing Jiancheng Bioengineering Institute, Nanjing, China), IL-1β (#ml098416), IL-6 (#ml098430), tumor necrosis factor (TNF)-α (#ml002095), and TLR4 (#ml037978) (Shanghai Enzyme-linked Biotechnology Co., Ltd., Shanghai, China) levels in mice, RAW264.7 cells were treated with tripdiolide and LPS, and then mixed with NS at a ratio of 1:10 (w: v). After homogenization, centrifuged at 10000 r/min to obtain the supernatant as the cell samples for testing. The protein concentration was quantified using the BCA assay kit (#23227, Thermo). The standard detection kits were employed for the quantification of ROS (#E004-1-1, Nanjing Jiancheng Bioengineering Institute), IL-1β (#ml098416), IL-6 (#ml098430) (Shanghai Enzyme-linked Biotechnology Co., Ltd.) levels in RAW264.7 cells.

### 2.6 Intestinal microbiota analysis

Cecal contents from four randomly selected mice in each group were analyzed to assess intestinal microbiota profiles. Genomic DNA samples of total intestinal microbiota were extracted using the OMEGA Soil DNA Kit (#D5625-01, OmegaBio-Tek, Norcross, GA, United States). Extracted DNA from cecal contents and performed PCR amplification with a specific focus on the V3-V4 region of 16SrRNA. Agencourt AMPure XP beads (Beckman Coulter, Indianapolis, IN, United States) were used to purify the PCR products, and a PicoGreen dsDNA Assay Kit was used for quantification (Invitrogen, Carlsbad, CA, United States). Sequencing was performed using the double ended method in Illumina MiSeq platform with a sequencing length of 2 × 250 bp, analysis was performed by Shanghai Parosen Biotechnology Co., Ltd. (Shanghai, China). The raw sequencing data is uploaded to NCBI SRA, which could be accessed through the website link (https://www.ncbi.nlm.nih.gov/sra/PRJNA1110827). DADA2 with a 100% similarity threshold was used for clustering sequencing data. The differences in amplicon sequence variation (ASVs) composition between groups were compared base on ASVs data. The Venn diagram is used to compare the number of shared microorganisms between groups and the number of unique microorganisms in each group. The evaluation indicators of alpha diversity (Pielou_e and Faith_PD indices) were used as *post hoc* tests to verify the significance of the differences. Linear discriminant analysis Effect Size (LEfSe) and a relative abundance heatmap at the genus level with high importance were used to identify differentially abundant species between groups. The metabolic and functional pathways connected to the altered intestinal microbiota in tripdiolide-treated mice were discovered by examining the metabolic pathways across all domains of life using the MetaCyc database (Liu et al., 2024b).

### 2.7 Western blotting

Kidney sample or RAW264.7 cells: RIPA Lysis Buffer (#20–188, Merck Millipore, Burlington, MA, United States) = 1:10 (w/v) mixture was homogenized to extract proteins. Protein levels were measured and leveled with BCA assay kit (#A55860, Thermo). The target protein underwent separation using a gel electrophoresis technique involving sodium dodecyl sulfate polyacrylamide gel with a concentration range of 10%–12%. Subsequently, the separated protein was transferred onto a PVDF membrane (0.45 μm, Thermo). The blocking solution (#GF1815, Culham Science Centre, Oxfordshire, United Kingdom) is applied to the membrane in a sequential manner, incubated with anti-nuclear factor erythroid 2-related factor 2 (Nrf2) (#20733, CST, MA, United States), anti-TLR4 (#ab217274, Abcam, MA, United States), anti-heme Oxygenase-1 (HO-1) (#A1346), anti-SOD1 (#A12537), anti-NF-κB (#A19653), anti-p-NF-κB (#AP0475), anti-NLRP3 (#A5652) (Abclonal, Wuhan, China), or anti-GAPDH (#E-AB-20032) antibodies, and incubated with Goat Anti-Rabbit (#E-AB-1003) or Goat-Anti-Mouse (#E-AB-1001) (Elabscience, Wuhan, China) antibodies. Multicolor Prestained Protein Ladder (#WJ103, Shanghai Yamay Biomedical Technology Co., Ltd., Shanghai, China) was used to label protein molecular weight. Electrochemiluminescence kits (#GK10006, GLPBIO, Montclair, CA, United States) was used for luminescent reactions, and imaging system (Tanon 5200, Tanon Science and Technology Co., Ltd., Shanghai, China) was used for detecting reactions. The quantification of pixel density was performed using ImageJ v1.8.0 (National Institutes of Health, Bethesda, MD, United States) (Zhou et al., 2024).

### 2.8 Statistical analysis

Differences were assessed using two-tailed Student’s t-test (DSS Statistics 25, BONC Technology Co., Ltd., Beijing, China). Statistical significance for the differences in data between groups was defined as a *p*-value less than 0.05.

## 3 Results

### 3.1 Effect of tripdiolide on STZ-induced diabetic mice

**FIGURE 1:**
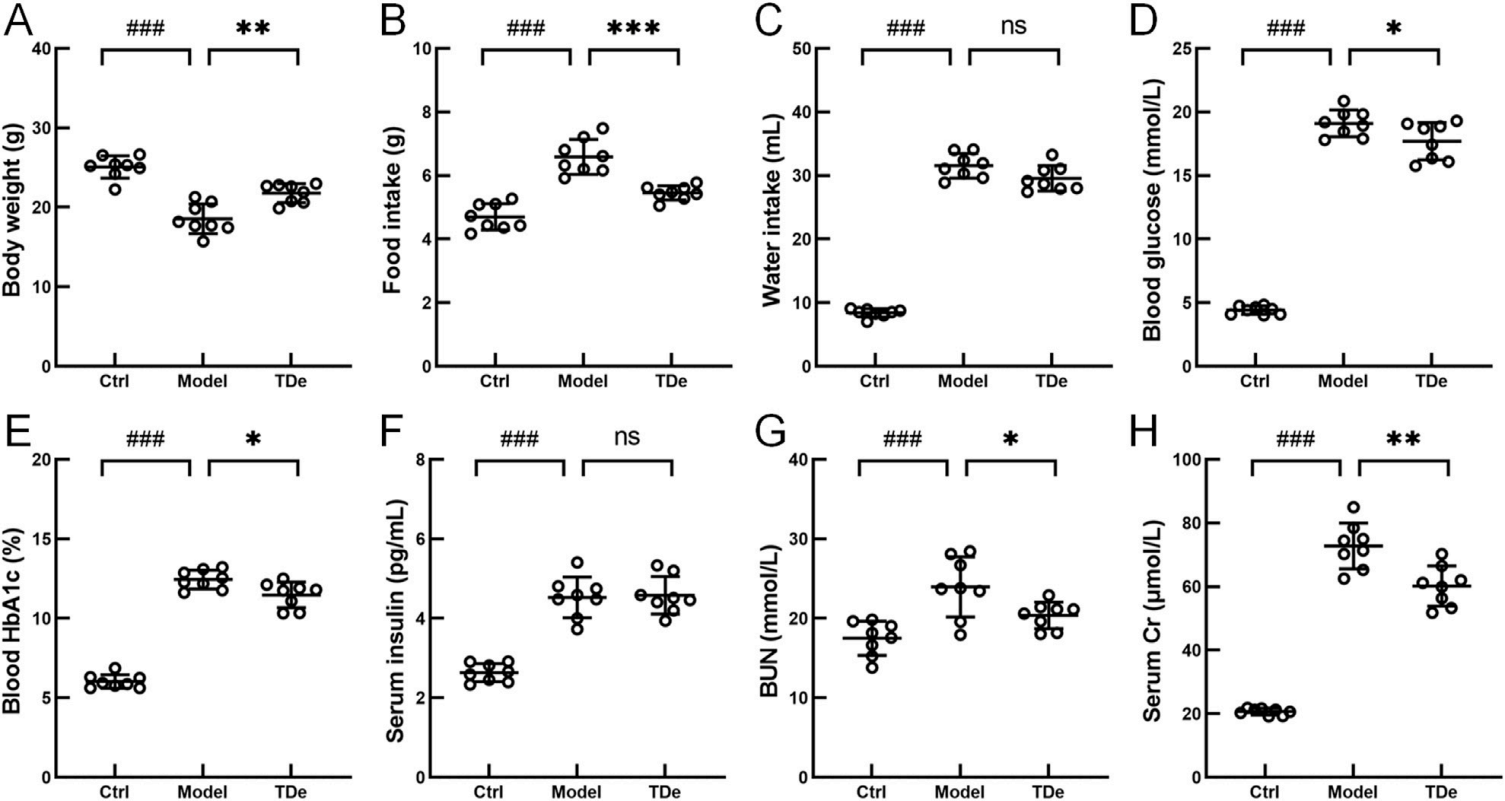
The effects of tripdiolide on diabetic mice. The changes of **(A)** Body weight, **(B)** food intake, **(C)** water intake, **(D)** blood glucose, **(E)** blood HbA1c, **(F)** serum insulin, **(G)** BUN, and **(H)** serum Cr levels (n = 8) in tripdiolide-treated diabetic mice. Data are shown as means ± SD, ^###^ *P* < 0.001 vs. Ctrl mice, *P < 0.05, **P < 0.01, ***P < 0.001 vs. Model mice. BUN, blood urea nitrogen; Cr, creatinine; TDe, tripdiolide.

To investigate the holistic impact of tripdiolide on diabetic mice, physiological and biochemical indices, including blood glucose and body weight, were assessed. After 10 weeks treatment, compared with Ctrl mice, the body weight decreased by 26% (*p* < 0.001) (Figure 1A), and the intake of food and water increased significantly (*p* < 0.001) (Figures 1B,C) in diabetic mice. Tripdiolide significantly increased the body weight by 17.4% (*p* < 0.01), reduced the food intake by 17.2% (*p* < 0.001), but fail to affect the reduction of water intake (*p* > 0.05) in diabetic mice (Figures 1A–C). Compared with Ctrl mice, the fasting blood glucose was markedly enhanced by 331.8% (*p* < 0.001), the blood HbA1c was significantly increased by 106.4% (*p* < 0.001), and the serum insulin was markedly increased by 71.8% (*p* < 0.001) in diabetic mice (Figures 1D–F). Tripdiolide markedly decreased blood glucose and blood HbA1c (*p* < 0.05), but did not yield any substantial impact on insulin level (*p* > 0.05) in diabetic mice (Figures 1D–F). Serum Cr and BUN in diabetic mice were markedly escalated by 251.5% and 37% (*p* < 0.001), respectively, which were reversed by tripdiolide treatment (*p* < 0.05) (Figures 1G–H).

**FIGURE 2:**
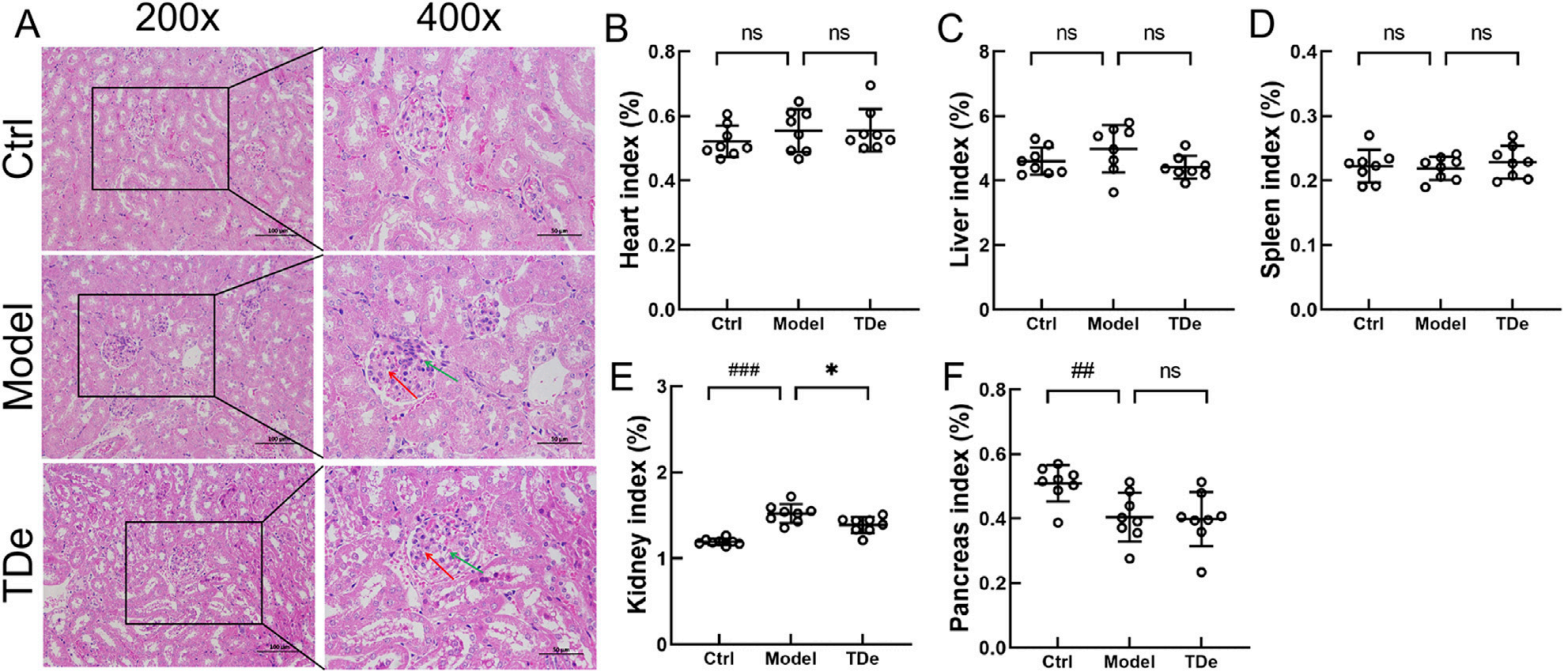
The effects of tripdiolide on organs of diabetic mice. **(A)** Histological changes in kidney (200×, Scale bar: 100 μm; 400×, Scale bar: 50 μm), and the changes of **(B)** heart, **(C)** liver, **(D)** spleen, **(E)** kidney, and **(F)** pancreas indices (n = 8) in tripdiolide-treated diabetic mice. Red arrow, extracellular matrix deposition. Green arrow, inflammatory cells. Data are shown as means ± SD, ^##^ *P* < 0.01, ^###^ *P* < 0.001 vs. Ctrl mice, *P < 0.05 vs. Model mice. TDe, tripdiolide.

Pathological changes are a visual evaluation method for kidney injury. Compared with Ctrl mice, diabetic mice displayed serve extracellular matrix deposition (histological score: 2.6) and significant inflammatory cell infiltration (histological score: 2.4) in glomerular, tripdiolide treatment reversed extracellular matrix deposition (histological score: 1.8) and significant inflammatory cell infiltration (histological score: 1.6) (Figure 2A; Table 1). There were no significant changes in heart, liver and spleen indices among three group mice (Figures 2B–D). The kidney index was markedly escalated by 27.4% (*p* < 0.001) and pancreas index was markedly decreased by 20.6% (*p* < 0.01) in diabetic mice (Figures 2E,F). Tripdiolide significantly reversed the kidney index increasing (*p* < 0.05), but did not yield any substantial impact on pancreas index (*p* > 0.05) in diabetic mice (Figures 2E–D). Tripdiolide demonstrated a pronounced protective effect on the kidney in diabetic mice, highlighting its superior efficacy in preserving kidney function.

### 3.2 Effect of tripdiolide on intestinal microbiota in diabetic mice

**FIGURE 3:**
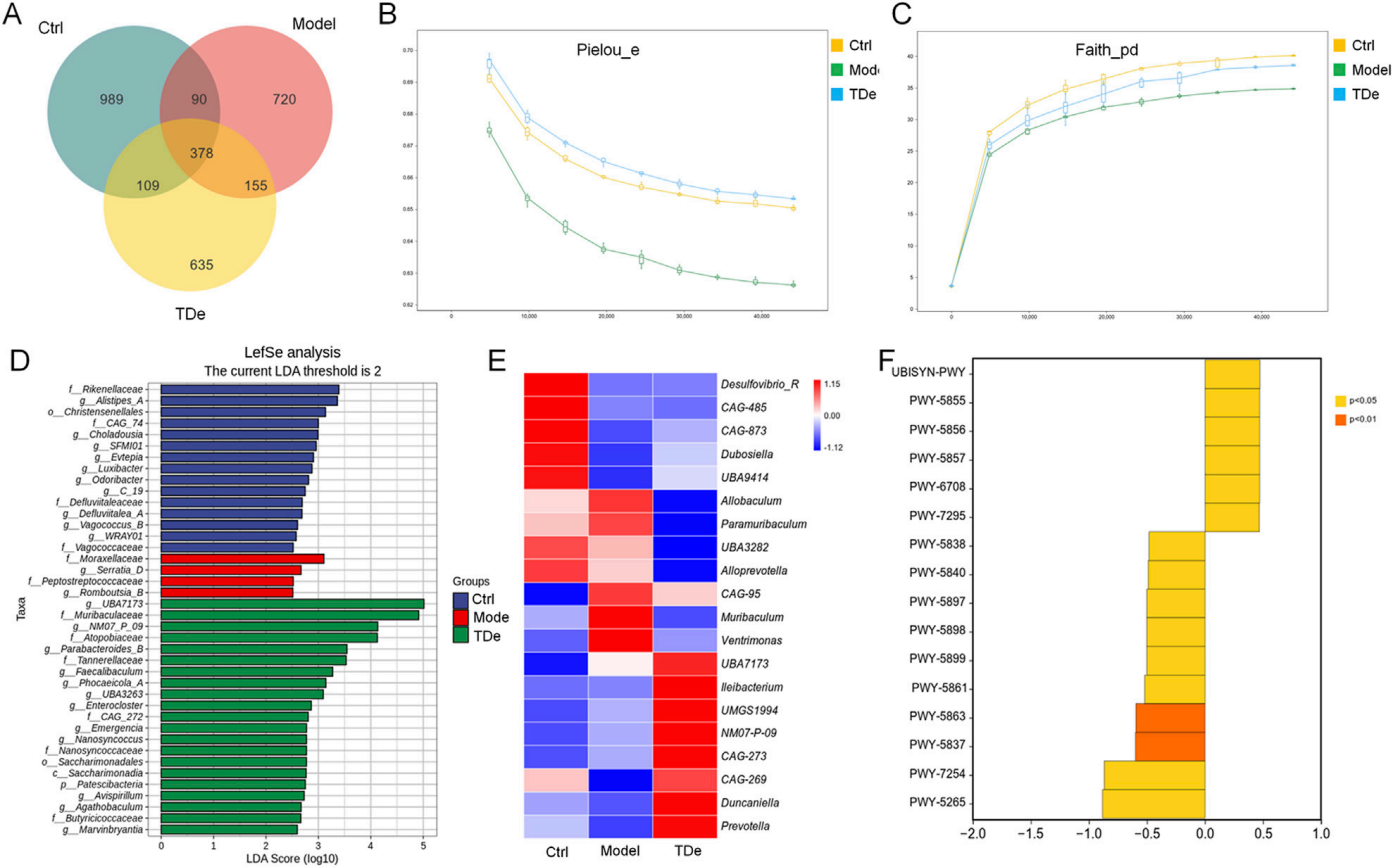
Regulation of tripdiolide on intestinal microbiota of diabetic mice. **(A)** Venn diagram of ASVs in the mice intestinal microbiota. Alpha diversity was analyzed by **(B)** Pielou_e, and **(C)** Faith_PD. **(D)** LEfSe analysis of the dominant biomarker taxa among the four groups. The current LDA threshold is 2. **(E)** Heatmap of the top 20 genera by average abundance. **(F)** Difference analysis of metabolic pathways between tripdiolide-treated mice and diabetic mice based on MetaCyc. TDe, tripdiolide.

The imbalance of intestinal microbiota directly or indirectly affects the occurrence and development of kidney diseases (Noel et al., 2014). A total of 3076 ASVs were detected in the cecal contents of all mice, among which 989, 720, and 635 were unique ASVs for Ctrl mice, diabetic mice, and Tripdiolide-treated mice, respectively. The total number of ASVs shared by three groups of mice was 378 (Figure 3A). Compared with Ctrl mice, the Pielou_e index and Faith_pd index of intestinal microbiota in diabetes mice decreased, and the Pielou_e index and Faith_pd index increased after tripdiolide treatment (Figures 3B,C). Tripdiolide treatment improved the evenness and diversity of intestinal microbiota in diabetes mice (Hsieh and Chao, 2017; Deng et al., 2022). The LEfSe is employed to discern the biomarkers within the intestinal microbiota of different mice, spanning from phylum to genus level. Four biomarkers (including *Serratia* and *Romboutsia* genera) were found in the intestinal microbiota of diabetes mice, and 21 biomarkers (including *Parabacteroides*, *Faecaalibacillus*, *Phocaeicola*, *Enterocluster*, *Nanosyncus*, *Avispirillum*, *Agathobacillus*, *Marvinbryantia* genera) were found in the intestinal microbiota of mice treated with tripdiolide (Figure 3D). The abundance differences of top 20 genera intestinal microbiota were compared by heatmap, tripdiolide decreased the abundance of *Allobaculum*, *Parameribaculum*, *Muribaculum*, *Yentrimonas* and *Alloprevotella*, and increased the abundance of *Dubosiella*, *Ileibacterium*, *Duncaniella* and *Prevotella* in diabetic mice (Figure 3E). The differences of metabolic pathways between tripdiolide-treated mice and diabetic mice were compared based on MetaCyc. Compared with diabetic mice, tripdiolide significantly increased 6 metabolic pathways including ubiquinol-7, 8, 9, 10 biosynthesis (prokaryotic), and decreased 10 metabolic pathways including superpathway of menaquinol-7, 8, 11, 12, 13 biosynthesis (Figure 3F; Table 2). Ubiquinol and menaquinol play a crucial part in the control of redox reaction process (Ernster and Forsmark-Andree, 1993; Suharti et al., 2001).

**TABLE 2:** Metabolic pathway with significant differences between tripdiolide-treated mice and diabetic mice based on MetaCyc.

Pathway	Description	logFC	SE	*P* values	adj*P* values
UBISYN-PWY	superpathway of ubiquinol-8 biosynthesis (prokaryotic)	0.4728	0.1398	0.000716	0.01899
PWY-5855	ubiquinol-7 biosynthesis (prokaryotic)	0.4711	0.14	0.000764	0.01899
PWY-5856	ubiquinol-9 biosynthesis (prokaryotic)	0.4711	0.14	0.000764	0.01899
PWY-5857	ubiquinol-10 biosynthesis (prokaryotic)	0.4711	0.14	0.000764	0.01899
PWY-6708	ubiquinol-8 biosynthesis (prokaryotic)	0.4711	0.14	0.000764	0.01899
PWY-5837	1,4-dihydroxy-2-naphthoate biosynthesis I	−0.5979	0.1388	1.65E-05	0.003195
PWY-5863	superpathway of phylloquinol biosynthesis	−0.5957	0.1386	1.72E-05	0.003195
PWY-5861	superpathway of demethylmenaquinol-8 biosynthesis	−0.5189	0.1395	0.0001996	0.01899
PWY-5897	superpathway of menaquinol-11 biosynthesis	−0.5012	0.1411	0.0003809	0.01899
PWY-5898	superpathway of menaquinol-12 biosynthesis	−0.5012	0.1411	0.0003809	0.01899
PWY-5899	superpathway of menaquinol-13 biosynthesis	−0.5012	0.1411	0.0003809	0.01899
PWY-7254	TCA cycle VII (acetate-producers)	−0.8679	0.2479	0.000464	0.01899
PWY-5840	superpathway of menaquinol-7 biosynthesis	−0.4879	0.1411	0.0005441	0.01899
PWY-5838	superpathway of menaquinol-8 biosynthesis I	−0.4812	0.1421	0.0007048	0.01899
PWY-5265	peptidoglycan biosynthesis II (staphylococci)	−0.8827	0.2624	0.0007678	0.01899

### 3.3 Impact of tripdiolide on oxidative stress and inflammation in the kidneys of mice with diabetes

**FIGURE 4:**
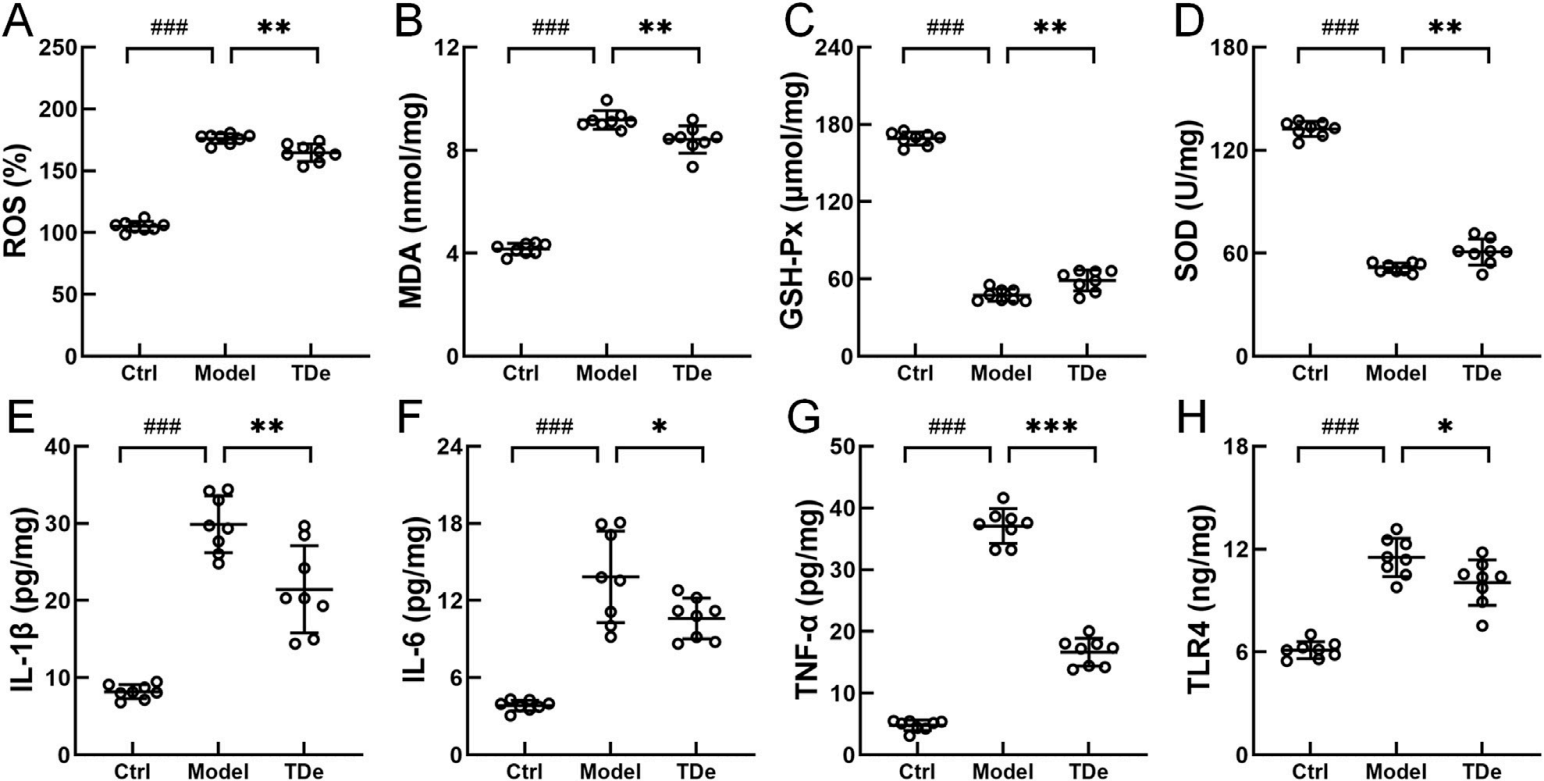
Regulation of tripdiolide on biochemical factors in kidney of diabetic mice. Tripdiolide treatment reduced the levels of **(A)** ROS, **(B)** MDA, increased the levels of **(C)** GSH-Px and **(D)** SOD, and reduced the expressions of **(E)** IL-1β, **(F)** IL-6, **(G)** TNF-α, and **(H)** TLR4 in kidney of diabetic mice. Data are shown as means ± SD, ^###^ *P* < 0.001 vs. Ctrl mice, *P < 0.05, **P < 0.01, ***P < 0.001 vs. Model mice. ROS, reactive oxygen species; MDA, malondialdehyde; GSH-Px, glutathione peroxidase; SOD, superoxide dismutase; IL-1β, interleukin-1β; IL-6, interleukin-6; TNF-α, tumor necrosis factor-α; TLR4, toll-like receptor 4; TDe, tripdiolide.

To investigate the protective effect of tripdiolide on kidney injury in diabetic mice and its association with oxidative stress and inflammation regulation, the levels of oxidative stress markers and inflammatory factors were measured. Compared with Ctrl mice, ROS and MDA in the kidney of diabetes mice were significantly increased by 67.6% and 120.4% (*p* < 0.001), respectively (Figures 4A,B). Tripdiolide treatment significantly decreased the levels of ROS and MDA in kidney of diabetes mice (*p* < 0.01) (Figures 4A,B). SOD and GSH-Px in the kidney of diabetes mice were markedly reduced by 61.0% and 71.9% (*p* < 0.001) respectively, which were significantly increased by over 17.7% (*p* < 0.01) after tripdiolide treatment (Figures 4C,D). Tripdiolide reduced the oxidative stress in diabetes mice kidney, which was consistent with intestinal microbiota results. Compared with Ctrl mice, IL-1β, IL-6, TNF-α and TLR4 in the kidney of diabetes mice both significantly increased by over 88.5% (*p* < 0.001), tripdiolide markedly decreased the levels of TNF-α, IL-1β, IL-6, TLR4 by 55.1% (*p* < 0.001), 28.2% (*p* < 0.01), 23.4% (*p* < 0.05), and 12.7% (*p* < 0.05), respectively (Figures 4E–H). The anti-inflammatory effects of tripdiolide in diabetic mouse kidneys were supported by renal pathology and intestinal microbiota analysis.

### 3.4 Effect of tripdiolide on Nrf2/NF-κb pathway

**FIGURE 5:**
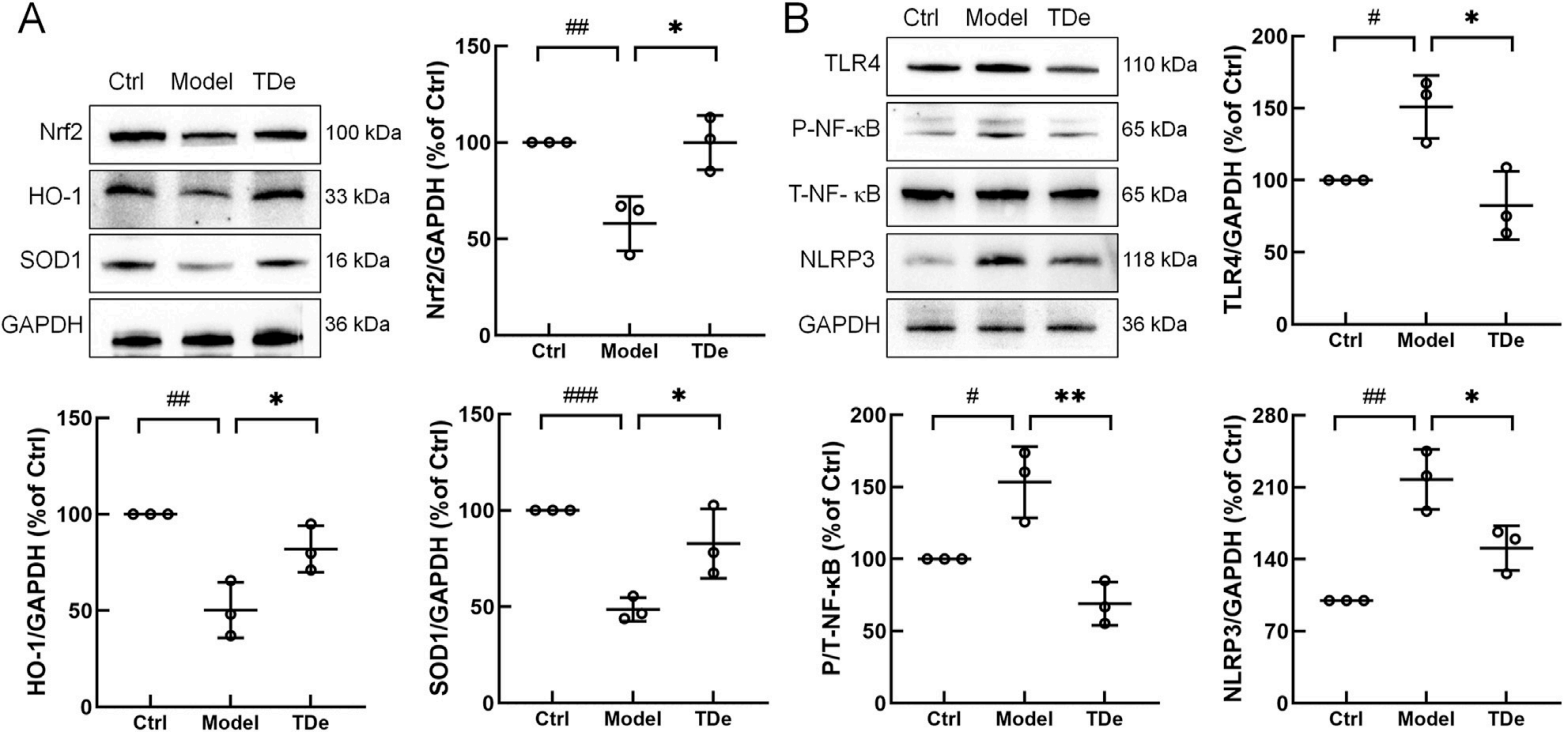
Tripdiolide activated the Nrf2 pathway and inhibited the NF-κB pathway, thus exerting the kidney protection effect. **(A)** Tripdiolide increased the expression levels of Nrf2, HO-1, SOD1, and **(B)** decreased the expression levels of TLR4, P-NF-κB, NLRP3 in kidney of diabetic mice. Quantification data were normalized by GAPDH and their corresponding total proteins and the corresponding protein expression of Ctrl mice was considered to be 100%. Nrf2, nuclear factor erythroid 2-related factor 2; HO-1, heme Oxygenase-1; SOD1, superoxide dismutase 1; TLR4, toll-like receptor 4; P-NF-κB, phosphorylated nuclear factor-κB; NLRP3, NOD-like receptor protein 3; TDe, tripdiolide.

To elucidate the potential mechanism underlying the renal protective effects of tripdiolide in diabetic mice, the expression levels of Nrf2/NF-κB pathway-related proteins were determined. Compared with Ctrl mice, the expression of Nrf2, HO-1 and SOD1 proteins in kidney of diabetes mice were significantly reduced (*p* < 0.01) (Figure 5A). Tripdiolide markedly escalated the expression of Nrf2 (by 72.2%), HO-1 (by 62.9%), and SOD1 (by 70.0%) in the kidney of diabetes mice (*p* < 0.05) (Figure 5A). Compared with Ctrl mice, the expression of TLR4, P-NF-κB and NLRP3 in the kidney of diabetes mice were markedly escalated (*p* < 0.01) (Figure 5A). Tripdiolide treatment significantly decreased TLR4 by 45.3%, P-NF-κB by 54.9%, and NLRP3 by 30.7% in the kidney of diabetes mice (*p* < 0.05) (Figure 5B).

**FIGURE 6:**
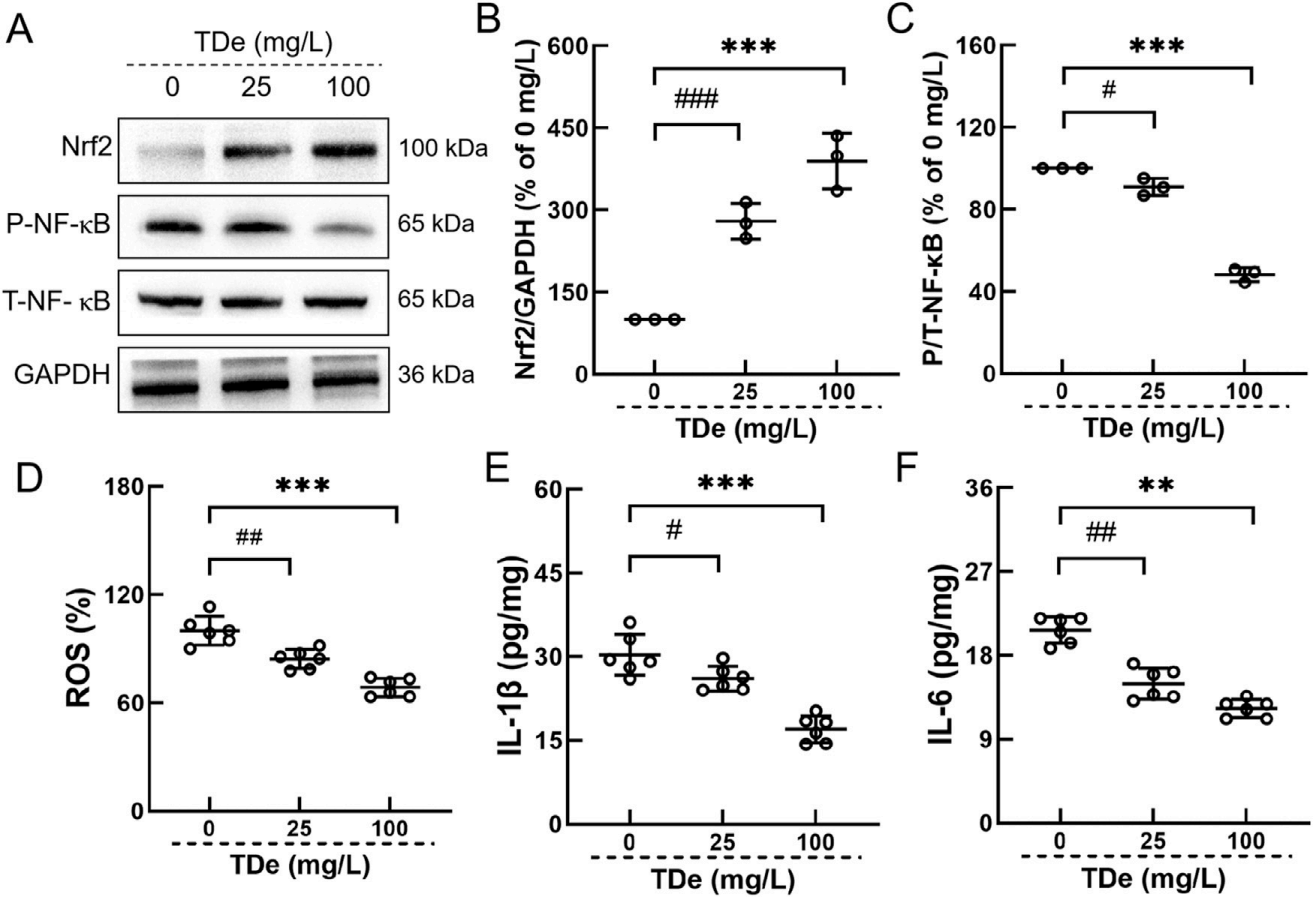
Tripdiolide activated the Nrf2 and inhibited the NF-κB in RAW264.7. **(A)** Tripdiolide increased the expression levels of Nrf2 and decreased the expression levels of P-NF-κB. Quantification data of **(B)** Nrf2 and **(C)** P-NF-κB. Tripdiolide treatment reduced the levels of **(D)** ROS, **(E)** IL-1β, **(F)** IL-6 in RAW264.7. Quantification data were normalized by GAPDH and their corresponding total proteins and the corresponding protein expression of Ctrl mice was considered to be 100%. Nrf2, nuclear factor erythroid 2-related factor 2; P-NF-κB, phosphorylated nuclear factor-κB; ROS, reactive oxygen species; IL-1β, interleukin-1β; IL-6, interleukin-6; TDe, tripdiolide.

In LPS-treated RAW264.7 cells model, 100 mg/L tripdiolide markedly escalated the expression of Nrf2 by 289.5%, and reduced the phosphorylation level of NF-κB by 51.7% (*p* < 0.001) (Figures 6A–C). Tripdiolide (100 mg/L) treatment significantly decreased the levels of ROS by 31.4%, IL-1β by 43.9%, IL-6 by 40.6% compared with LPS-treated RAW264.7 cells (*p* < 0.001) (Figures 6D–F). Cell experiment results effectively recapitulates key aspects of diabetes observed in mice. Nrf2/NF-κB pathway plays a crucial role in tripdiolide-mediated protection against diabetic kidney injury.

## 4 Discussion

In this study, tripdiolide preventive treatment effectively attenuated hyperglycemia in diabetic mice, ameliorated kidney pathology, and notably decreased serum Cr and BUN. The preventive treatment of tripdiolide also modulated the abundance of various intestinal microorganisms, while its distinct metabolic pathway primarily targeted oxidative stress-related processes. This intervention regulated induction of the Nrf2/NF-κB signaling pathway in the kidney tissues of mice with diabetes, thereby mitigating diabetes-induced kidney injury by modulation of oxidative stress and inflammatory processes.

Dysbiosis of the intestinal microbiota frequently manifests in a range of autoimmune and metabolic disorders, including inflammatory bowel disease, chronic kidney disease, diabetes, and obesity (Carding et al., 2015; Lau and Vaziri, 2017). Patients with diabetic nephropathy often exhibit alterations in the formation of their intestinal microbiota, and a diverse range of intestinal microbial communities plays a role in the onset and advancement of diabetic kidney disease as well as other related disorders (Cheng et al., 2024). The preventive treatment of tripdiolide leaded to a decrease in abundance of *Allobaculum*, while increasing the abundance of *Dubosella* and *Prevotella* in diabetic mice. *Allobaculum* has been found to exhibit a direct association with uremic metabolites in obese mice, thereby exerting an impact on the systemic inflammatory response and the development of inflammatory bowel disease (Barouei et al., 2017). *Dubosiella* exhibits anti-inflammatory properties in colitis and is intimately related to regulating iron death processes (Gu et al., 2023). Supplementation with *Dubosiella* effectively enhances the composition of intestinal microbiota in mice and inhibits oxidative stress by elevating superoxide dismutase (SOD) activity (Liu et al., 2023). Dietary fiber supplementation increases the abundance of *Prevotella* in the intestines of diabetic mice, promoting the synthesis of short-chain fatty acids and thereby exerting a protective effect against the development and progression of diabetic kidney disease (Li et al., 2020). Based on MetaCyc analysis of intestinal microbiota, the differential metabolic pathways influenced by tripdiolide are predominantly associated with the biosynthesis of ubiquinol and menaquinol. Ubiquinol, a reduced form of coenzyme Q10, and menaquinol, a reduced form of vitamin K2, serve as critical substrates in cellular redox reactions, playing essential roles in electron transport and energy metabolism (Rothery et al., 2001; Thesseling et al., 2019). These suggested that tripdiolide may exert renoprotective effects on diabetic nephropathy by modulating intestinal microbiota composition and reducing systemic oxidative stress levels, thereby potentially mitigating its occurrence and progression.

Prolonged hyperglycemia induces the generation of ROS, can cause harm to large molecules like lipids, carbohydrates, proteins, and nucleic acids, leading to the development of kidney dysfunction in the long run (Sifuentes-Franco et al., 2018). Continuous ROS can also lead to NF-κB activation and NLRP3 transcription, thereby activating the expression of inflammatory factors (Kelley et al., 2019; Swanson et al., 2019). The excessive generation of ROS and the subsequent induction of inflammation, fibrosis, and endothelial dysfunction constitute primary etiological factors contributing to kidney injury in diabetes (Simon et al., 2000; Singh et al., 2011). The interconnectedness of oxidative stress and inflammation is indissoluble in both physiological and pathological scenarios. This intricate interaction gives rise to a self-perpetuating feedback loop, thereby perpetuating a deleterious cycle (Wu et al., 2020). Consequently, ameliorating oxidative stress and inflammation can effectively ameliorate various complications associated with diabetes, including kidney disease (Tanase et al., 2022). In this study, tripdiolide treatment exhibited a noteworthy reduction in ROS and MDA, increased antioxidant enzymes, upregulated Nrf2, HO-1, and SOD1. The activation of Nrf2 can elicit the upregulation of HO-1 and SOD1, leading to enhanced antioxidant enzyme activity and reduced ROS, thereby ameliorating oxidative stress in diabetic nephropathy, which makes a contribution to the attenuation of diabetes-associated kidney fibrosis and ultimately serves as a preventive measure against diabetic nephropathy (Huang et al., 2015). In the initial stages of diabetic nephropathy, Nrf2 function is significantly impaired, resulting in an imbalance of oxidative-reduction. Activation of Nrf2 can effectively alleviate diabetic nephropathy (Mohan et al., 2020). Tripdiolide treatment inhibited the level of oxidative stress in diabetes mice, and the findings aligned with the outcomes obtained from analyzing intestinal microbiota.

NF-κB activation is subject to inhibition by Nrf2, both directly and indirectly. Upon entering the nucleus, Nrf2 promotes the expression of downstream antioxidant enzymes and reduced ROS, a key activator of NF-κB (Morgan and Liu, 2011; Shen et al., 2022). Nrf2 may directly inhibit the activation of NF-κB by modulating the phosphorylation of the IKK complex via mitogen-activated protein kinase family (Bellezza et al., 2010). Overexpression of Nrf2 results in decreased levels of TLR4, NF-κB, and downstream inflammatory cytokines such as IL-1β and TNFα (Li et al., 2023). The administration of tripdiolide in this study resulted in a notable decrease of IL-1β, IL-6, TNF-α, and TLR4, while also inhibiting TLR4, P-NF-κB, and NLRP3 in diabetic mice kidneys. Hyperglycemia can promote the activation of TLR4, NF-κB pathway and NLRP3, and the subsequent inflammatory and fibrosis reactions, resulting in the emergence of diabetic nephropathy (Ma et al., 2014). The activation of the NF-κB pathway and NLRP3 can enhance various inflammatory mediators, including IL-6, TNF-α, and IL-1β (Lin et al., 2014; Aboismaiel et al., 2024). Reactive overproduction of proinflammatory cytokines facilitates the progression of diabetes-associated inflammation and endothelial dysfunction (Lopez-Bojorquez et al., 2004; Oliveira et al., 2008). The administration of tripdiolide effectively attenuated kidney inflammation in diabetic mice, as indicated by reduced inflammatory cell infiltration observed in the histopathological analysis. The effect of tripdiolide on alleviating kidney injury in diabetes is related to regulating Nrf2/NF-B pathway, reducing oxidative stress and inflammation.

Diabetic nephropathy is one of the leading causes of end-stage renal disease and continues to lack a definitive cure (Paul et al., 2023). Although certain therapeutic agents, such as angiotensin-converting enzyme (ACE) inhibitors and sodium-dependent glucose transporters 2 (SGLT2) inhibitors, have shown promise in managing the complications of diabetic nephropathy, their clinical utility is often questioned due to poor solubility, low bioavailability, inability to halt disease progression despite symptom alleviation, and low patient adherence (Paul et al., 2023; Amanat et al., 2025; Cherney et al., 2025). Traditional approaches are largely reactive, failing to target early pathological mechanisms. Preventive strategies could interrupt kidney damage during the latent phase, enabling proactive and personalized solutions. Tripdiolide demonstrated little influence on blood glucose, glycated hemoglobin, and insulin levels in this study, pointing to its limited efficacy in glycemic management. Instead, its key function seems to lie in protecting the kidneys from damage triggered by hyperglycemia. Therefore, triptolide may need to be combined with blood glucose management strategies in clinical practice. Translating these findings into clinical use is hindered by various limitations, including possible off-target effects and long-term safety concerns, highlighting the need for further development.

Our study had certain limitations. The precise mechanisms through which tripdiolide confers renal protection and modulates the microorganisms remain poorly understood. Furthermore, the study was limited to a single dosage of tripdiolide, leaving the dose-response relationship unexplored. Further research is needed to elucidate these mechanisms and to establish the optimal dosing regimen for therapeutic efficacy.

## 5 Conclusion

In summary, tripdiolide may protect against hyperglycemia-induced kidney injury by modulating the Nrf2/NF-κB pathway through intestinal microbiota regulation, thereby alleviating oxidative stress and inflammation. This study provides theoretical support for the potential of tripdiolide in treating and preventing diabetic nephropathy.

## Data Availability

The datasets presented in this study can be found in online repositories. The names of the repository/repositories and accession number(s) can be found below: https://www.ncbi.nlm.nih.gov/, PRJNA1110827.
